# A Survey: Who Manages the Diabetic Foot in India?

**DOI:** 10.1055/s-0045-1814164

**Published:** 2026-01-05

**Authors:** Pawan Agarwal, Dhananjaya Sharma

**Affiliations:** 1 Department of Plastic Surgery, Netaji Subhash Chandra Bose Government Medical College, Jabalpur, Madhya Pradesh, India; 2 Department of Surgery, Netaji Subhash Chandra Bose Government Medical College, Jabalpur, Madhya Pradesh, India

**Keywords:** diabetic foot, India, surgical specialties, survey, plastic surgery, orthopaedic surgeons, data gaps, diabetic foot clinics, nerve transfers

## Abstract

**Introduction:**

Diabetic foot is a major cause of morbidity and nontraumatic amputations in India. Despite national guidelines, care remains fragmented across specialties. This study aimed to examine the roles of various surgical specialists in diabetic foot care in India.

**Materials and Methods:**

A web-based survey comprising 20 questions was distributed via WhatsApp to general, plastic, orthopaedic, and vascular surgeons across India. The survey assessed specialty involvement, interdisciplinary collaboration, procedural roles, availability of protocols, and awareness of newer techniques. Responses were analyzed using descriptive statistics.

**Results:**

A total of 126 surgeons responded: 50% were plastic surgeons, followed by general (25%), orthopaedic (16%), and vascular surgeons (9%). While 94% favored dedicated diabetic foot clinics, only 16% reported the presence of written institutional protocols. Plastic surgeons were the most commonly identified team leaders (83%) and were predominantly involved in wound coverage (97%). Vascular surgeons led in revascularization (86%) and orthopaedic surgeons in deformity correction (62%). Collaboration was highest with physicians (79%). Awareness of nerve transfer techniques was high (86%), but practice was limited.

**Conclusion:**

The survey highlights strong support for multidisciplinary diabetic foot care but reveals widespread gaps in structured protocols and coordinated team roles. A national call to action is needed to implement unified, context-sensitive, team-based diabetic foot care pathways across health care levels.

## Introduction

Diabetic foot is defined by the World Health Organization as “ *The foot of a diabetic patient that has the potential risk of pathologic consequences, including infection, ulceration, and/or destruction of deep tissues associated with neurologic abnormalities, various degrees of peripheral vascular disease, and/or metabolic complications of diabetes in the lower limb* .” 1 The diabetic foot is a leading cause of hospital admissions and nontraumatic lower limb amputations in India. 2 3 Despite the availability of guidelines for integrated diabetic foot care, actual practice patterns and specialty involvement vary widely across institutions. There are little published data on the roles played by various surgical specialties in the management of the diabetic foot in India. This survey explores the current landscape of diabetic foot management across Indian hospitals and surgical units.

## Materials and Methods

A web-based questionnaire was developed using Google Forms. The questionnaire contained 17 questions exploring roles of different specialties in diabetic foot teams, collaboration among specialties, timing and type of surgical involvement, availability of written protocols, and knowledge of newer interventions such as nerve transfers ( Supplementary Material 1 , available in the online version). The study was approved by the institutional ethics committee (Reference no: 6714/ 2025)

WhatsApp Messenger (Meta Platforms, Inc.) was used to distribute the questionnaire to plastic, orthopaedic, vascular, and general surgeons working in public and private hospitals in India (using contact lists of authors and lists of various surgical associations, and recipients were requested to further disseminate the survey to their contacts). The survey was open for 1 month (from May 1 to June 1, 2025) and reminder messages were sent after 2 and 3 weeks. Data were imported into an Excel spreadsheet and analyzed using SPSS 21.0 software. Descriptive statistics were used to analyze response distributions. Specialty-wise trends were noted. The categorical variables were expressed as numbers and percentages.

## Results

A total of 126 surgeons responded; 102 (81%) were male and 24 were female (19%). In addition, 29% were >50 years of age; 27% were between 41 and 50 years, 38% were between 31 and 40 years of age, and 6% between 20 and 30 years. Furthermore, 67% were working in teaching hospitals and 33% were in private hospitals. Among the responders, 23% were from corporate hospitals, 44% from medical colleges, and 33% from private clinics. Geographically, 50% responders were from central India, 30% from north India, and the remaining 20% from western India.

The majority were plastic surgeons (50%), followed by general surgeons (25%), orthopaedic surgeons (16%), and vascular surgeons (9%).

Survey responses are shown in Table 1 . Some salient points stood out:

- Need for specialized clinics: majority (94%) believe that diabetic foot should be treated in organized special clinics.
- Team leadership: 83% of respondents believed plastic surgeons should lead the diabetic foot team, with general surgeons (11%) as the next most common choice.
- Collaboration patterns: respondents reported most frequent collaboration with physicians (79%), plastic surgeons (62%), and vascular surgeons (51%).
- Timing of surgical involvement: plastic surgeons were most often involved for wound coverage (82%), whereas vascular surgeons were consulted mainly when ischemia was suspected (89%). Orthopaedic surgeons were commonly involved when osteomyelitis (68%) or deformity correction (43%) was required.
- Procedures by specialty: plastic surgeons performed a broad range of procedures, including wound coverage (97%), debridement (59%), and minor amputations (54%). Vascular surgeons primarily performed revascularization (86%), while orthopaedic surgeons were involved in deformity correction (62%) and major amputations (57%).
- System gaps: only 16% of respondents had a written local policy for acute diabetic foot sepsis management. However, 97% agreed that clear distribution of work among specialties should be specified, and 100% advocated for multidisciplinary collaboration.
- Awareness of advanced techniques: 49% reported performing internal offloading procedures; however, 86% were aware of nerve transfer as a modality to restore sole sensation.

**Table 1:** Summary of survey responses

S. No.	Question		Response
1	Who should lead the team for diabetic foot?	Plastic	83%
		Vascular	3%
		Orthopaedic	3%
		Gen surgeons	11%
2	Collaboration with other specialists	Ortho	48%
		Plastic	62%
		Vascular	51%
		Physician	79%
3	Do you involve plastic surgeons or VASCULAR surgeons when arterial disease is suspected?	Always	78%
		Sometimes	22%
		Never	0%
4	Which other clinical specialists attend your clinics?	Diabetes physicians	92%
		Microbiologists	30%
		Podiatrists	14%
		Orthotist	21%
5	At what stages of management do PLASTIC SURGEONS normally become involved with patients?	Initial assessment in the foot clinic	38%
		Urgent treatment of foot sepsis	33%
		Only when arterial disease is suspected	22%
		When wound coverage is required	82%
6	At what stages of management do ORTHOPAEDIC surgeons normally become involved with patients?	Initial assessment in the foot clinic	21%
		Urgent treatment of foot sepsis	8%
		When osteomyelitis is suspected	68%
		Only for deformity correction	43%
7	At what stages of management do VASCULAR SURGEONS normally become involved with patients?	Initial assessment in the foot clinic	19%
		Urgent treatment of foot sepsis	3%
		Only for deformity correction	0%
		When ischemia is suspected	89%
8	Operations for acute diabetic foot sepsis done by	Plastic surgeon	78%
		Vascular surgeons	5%
		Orthopaedic surgeons	19%
9	Which procedures do VASCULAR surgeons typically perform in patients with diabetic foot problems in your locality?	Abscess drainage	8%
		Debridement	19%
		Minor amputation	14%
		Major amputation	13%
		Correction of deformity	2%
		Wound coverage	6%
		Revascularization	86%
10	Which procedures do ORTHOPAEDIC surgeons typically perform in patients with diabetic foot problems in your locality?	Abscess drainage	22%
		Debridement	35%
		Minor amputation	41%
		Major amputation	57%
		Deformity correction	62%
		Wound coverage	2%
		Revascularization	0%
11	Which procedures do PLASTIC SURGEONS typically perform in patients with diabetic foot problems in your locality?	Abscess drainage	30%
		Debridement	59%
		Minor amputation	54%
		Major amputation	44%
		Deformity correction	48%
		Wound coverage	97%
		Revascularization	35%
12	Is there a written local policy in your hospital/clinic about the pathway of care for acute diabetic foot sepsis?	Yes	16%
		No	84%
13	Do you think the distribution of work to different specialties should be specified?	Yes	97%
		No	3%
14	Do you think there should be collaborations between different specialties to treat diabetic foot?	Yes	100%
		No	0%
15	Should diabetic foot be treated in organized special clinics?	Yes	94%
		No	6%
16	In your hospital, do you perform internal off-loading procedures?	Yes	49%
		No	51%
17	Do you know that lost sensation on the sole can be restored in diabetic foot by nerve transfer?	Yes	86%
		No	13%
		Not possible	1%

## Discussion

This national survey sheds light on the current practices and gaps in diabetic foot care in India. It highlights both the enthusiasm for multidisciplinary approaches and the uneven implementation of structured care pathways. The overwhelming majority of respondents advocated for collaborative care and the establishment of specialized diabetic foot clinics, yet only a minority reported the existence of formal local protocols.

The diabetic foot imposes a devastating burden worldwide. 4 Up to one-third of people with diabetes develop a foot ulcer in their lifetime, with recurrence rates exceeding 60% within 3 years of healing. Half of these ulcers become infected—leading to osteomyelitis in one in five cases—and nearly 20% of moderate or severe infections culminate in lower limb amputation. Strikingly, every 20 seconds, someone loses a leg to diabetes-related complications, with 85% of amputations preceded by a foot ulcer. Charcot neuro-arthropathy, though less common, adds further morbidity through destructive joint damage. Beyond the high mortality—more than double the 5-year risk of death compared with diabetes without ulcers—the economic and social costs are profound, encompassing health care expenditures, lost productivity, and caregiver strain. Despite this immense impact, data gaps persist due to inconsistent definitions and inadequate surveillance—particularly in low- and middle-income countries. 5 In India, the diabetic foot crisis is further intensified by late presentation, more advanced disease than global counterparts, and limited care infrastructure—contributing to 80% of all nontraumatic amputations nationwide. 2 3 These patterns reflect a pressing need for earlier intervention and strengthened diabetic foot care systems across the country.

Evidence shows that multidisciplinary teams ensure timely and integrated attention to glycemic control, wound care, vascular status, and infection, supported by clear care algorithms and referral pathways. 6 7 While more diabetic foot clinics are being run globally, there is variability in specialist participation, underscoring the need for more consistent clinic availability and better-defined pathways to maximize the benefits of interdisciplinary care. 8 Awareness of newer advances, like nerve transfer for re-innervation of the sole, is crucial to ensure that patients are not deprived of such treatment. 9 Similarly, in the context of diabetic foot complications, the phrase “ *time is tissue* ” emphasizes the critical importance of prompt intervention to prevent limb loss and potentially life-threatening situations. 10

India currently lacks widespread implementation of a unified, evidence-based approach to diabetic foot care, despite the Government of India having published standard treatment guidelines. 1 In practice, management remains fragmented across multiple specialists—physicians, surgeons (general, orthopaedic, plastic, vascular), endocrinologists, and podiatrists—with poorly defined roles and inconsistent protocols. This lack of coordination, combined with significant variability in health care access and delivery, undermines both prevention and treatment efforts. To address this growing public health challenge, it is imperative to operationalize and adapt the national guidelines into an integrated, context-specific framework that ensures comprehensive and coordinated diabetic foot care across the country.

As with all online surveys, this study is subject to several limitations. It includes the inability to calculate the actual percentage of responses, as it was disseminated openly. There is a possibility of a nonresponse and recall bias, with nonresponding surgeons being those who do not treat diabetic foot actively. Response bias may have favored those with a special interest in diabetic foot care. Specialty representation was uneven, and the voluntary nature of participation introduces self-selection bias. Survey fatigue may have affected nonresponders. The prominence of plastic surgeons in the survey responses—and in perceived leadership roles—likely reflects the respondent demographic, where half of the participants were from plastic surgery. This specialty-skewed sample is an important limitation and may have influenced the relative weighting of specialty roles, potentially underrepresenting the contributions of vascular and orthopaedic surgeons in many institutions. This survey is a pilot project to validate the questionnaire and a widespread survey is required to get responses from a larger and more diverse group of respondents. The result of the survey cannot be generalized nationwide with a small sample size and all regions were not represented in the study. These findings may be further validated by larger studies and future surveys should aim for a more balanced specialty representation. Despite these limitations, the survey provides valuable insights into the current state of diabetic foot care delivery in India and underscores the need for standardized, interdisciplinary protocols.

Our survey leads to a policy-oriented call to action:

- Institutionalize multidisciplinary diabetic foot units—particularly in teaching and tertiary centers—involving general, plastic, vascular, and orthopaedic surgeons, as well as physicians, podiatrists, and wound care nurses.
- National surgical and diabetic societies (e.g., Association of Surgeons of India, Diabetic Association of India) should collaborate to further refine available guidelines into more context-relevant, tiered protocols suited to various health care levels (primary to tertiary).
- Institutional policies should clearly define when and how each specialty is involved, especially in ischemia, osteomyelitis, wound reconstruction, and amputations.
- Other suggestions include audit and quality improvement, education (inclusion in teaching curricula for doctors and nurses), and capacity building, especially in tier II/III cities, to bridge the policy–practice gap.

## Conclusion

This survey highlights an urgent need for structured, multidisciplinary coordination across specialties for diabetic foot management in India. While most respondents agree on the necessity of collaborative care, specialized clinics, and clearer delineation of roles, formal pathways and institutional policies remain grossly lacking. The dominance of plastic surgery voices in this survey reflects both their increasing involvement in diabetic limb salvage and the need to broaden discourse to include underrepresented specialties. A paradigm shift is needed—one that transitions from fragmented specialty-led care to integrated, protocol-driven team models rooted in context-specific realities.
