# Supplementary material for: A Survey: Who Manages the Diabetic Foot in India?
Source: Indian J Plast Surg. 2026 Jan 5;59(3):186–90. doi: 10.1055/s-0045-1814164 (PMC13467271; doi:10.1055/s-0045-1814164)
Supplement: Supplementary file 1 [file 10-1055-s-0045-1814164-s2583685.pdf]

Supplementary Material

A Surgical Services Survey invitation: Who Manages the Diabetic Foot?

Diabetic foot complications are rising rapidly—and how we organize care matters. This brief survey explores how different surgical specialties currently contribute to diabetic foot care and aims to map existing service models across institutions and regions.

🕐 **It takes just 5 minutes**
🔒 **All responses are completely anonymous**
📊 **Findings will be shared in aggregate only**

Your insights are vital to understanding gaps and opportunities in diabetic foot care delivery. Please click the link below to participate:

https://forms.gle/mHvZUq6M6qLkQL6v8

If you have any questions, feel free to contact Dr. Pawan Agarwal at **drpawanagarwal@yahoo.com**.

Thank you for supporting this initiative.

Warm regards

1. Age
   20-30 years
   31-40 years
   41-50 years
   >50 years

2. Gender
   Male
   Female
3. years of practice
   <5 years
   5-10 years
   >10 years
4. your specialty
   Plastic surgeon
   Vascular surgeons;
   Orthopaedic surgeons;
   Gen surgery
   Others (please specify).

5. Do you treat diabetic foot infections?

a. Yes
b. No

If No, then answer no more questions and return the survey – thank you.

6. Which is the main hospital where you do see diabetic foot patients?
   Private clinic
   Corporate hospital
   Govt hospital
   Medical college

7. How many days in a week you run diabetic foot clinic?
   One day
   2 days
   3 days
   6 days

8. Do you consider the frequency of your diabetic foot clinics adequate?
   Yes
   No

9. According to you who should lead the team for diabetic foot management?
   Plastic surgeon
   Vascular surgeons;
   Orthopaedic surgeons;
   Gen surgeons
   Others (please specify).

10. Does your diabetic foot service involve collaboration with other specialists? If so, which are they?
    Ortho
    Plastic
    Vascular
    Physician
    None

11. Do you involve plastic surgeon or VASCULAR surgeons when arterial disease suspected?
    Always
    Sometimes
    Never

12. Which other clinical specialists attend your clinics?
    Diabetes physicians;
    Microbiologists;
    Podiatrists;
    Orthotists;

Other (please specify).

13. At what stages of management do PLASTIC SURGEONS normally become involved with patients?
Initial assessment in foot clinic;
Urgent treatment of foot sepsis;
Only when arterial disease suspected;
When wound coverage is required
Never

14. At what stages of management do ORTHOPAEDIC surgeons normally become involved with patients?
Initial assessment in foot clinic;
Urgent treatment of foot sepsis;
When osteomyelitis is suspected
Only for deformity correction;
Never

15. At what stages of management do VASCULAR SURGEONS normally become involved with patients?
Initial assessment in foot clinic;
Urgent treatment of foot sepsis;
Only for deformity correction;
When ischemia is suspected
Never

16. In your service, are operations for acute diabetic foot sepsis done by:
Plastic surgeon
Vascular surgeons;
Orthopaedic surgeons;
Others(please specify).

17. Which procedures do VASCULAR surgeons typically perform in patients with diabetic foot problems, in your locality?
Abscess drainage;
Debridement
Minor amputation
Major amputation (below knee/above knee);
Surgery to correct deformity;
Wound coverage
Revascularization

18. Which procedures do ORTHOPAEDIC surgeons typically perform in patients with diabetic foot problems, in your locality?
Abscess drainage;

Debridement
Minor amputation
Major amputation (below knee/above knee);
Surgery to correct deformity;
Wound coverage
Revascularization

19. Which procedures do PLASTIC SURGEONS typically perform in patients with diabetic foot problems, in your locality?
Abscess drainage;
Debridement
Minor amputation
Major amputation (below knee/above knee);
Surgery to correct deformity;
Wound coverage
Revascularization

20. Is there a written local policy in your hospital/clinic about the pathway of care for acute diabetic foot sepsis, and who deals with patients?
Yes
No.

21. Do you think distribution of work to different specialties should be specified?
Yes
No.

22. Do you think there should be collaborations between different specialties to treat diabetic foot?
Yes
No.

23. Diabetic foot should be treated in organized special clinics?

Yes
No

24. In your hospital do you perform internal off loading procedures?
Yes
No
25. Do you know lost sensation on sole can be restores in diabetic foot by nerve transfer?
Yes
No
Not possible
